# Barriers, facilitators, and priority needs related to cancer prevention, control, and research in rural, persistent poverty areas

**DOI:** 10.1007/s10552-023-01756-1

**Published:** 2023-08-01

**Authors:** Emily Hallgren, Karen H. K. Yeary, Peter DelNero, Beverly Johnson-Wells, Rachel S. Purvis, Ramey Moore, Stephanie Loveless, Kristen Shealy, Pearl A. McElfish

**Affiliations:** 1 College of Medicine, University of Arkansas for Medical Sciences Northwest, 2708 S. 48th St., Springdale, AR 72762 USA; 2 Department of Cancer Prevention and Control, Roswell Park Comprehensive Cancer Center, Buffalo, NY USA; 3 UAMS Regional Programs, University of Arkansas for Medical Sciences, West Helena, AR USA; 4 College of Medicine, University of Arkansas for Medical Sciences, Little Rock, AR USA

**Keywords:** Cancer health disparities, Persistent poverty, Rural, Qualitative, Cancer health equity

## Abstract

**Purpose:**

The purpose of this study was to identify the barriers, facilitators, and priority needs related to cancer prevention, control, and research in persistent poverty areas.

**Methods:**

We conducted three focus groups with 17 providers and staff of primary care clinics serving persistent poverty areas throughout the state of Arkansas.

**Results:**

We identified multiple barriers, facilitators, and priority needs related to cancer prevention and control at primary care clinics serving persistent poverty areas. Barriers included transportation, medical costs, limited providers and service availability, and patient fear/discomfort with cancer topics. Facilitators identified were cancer navigators and community health events/services, and priority needs included patient education, comprehensive workflows, improved communication, and integration of cancer navigators into healthcare teams. Barriers to cancer-related research were lack of provider/staff time, patient uncertainty/skepticism, patient health literacy, and provider skepticism/concerns regarding patient burden. Research facilitators included better informing providers/staff about research studies and leveraging navigators as a bridge between clinic and patients.

**Conclusion:**

Our results inform opportunities to adapt and implement evidence-based interventions to improve cancer prevention, control, and research in persistent poverty areas. To improve cancer prevention and control, we recommend locally-informed strategies to mitigate patient barriers, improved patient education efforts, standardized patient navigation workflows, improved integration of cancer navigators into care teams, and leveraging community health events. Dedicated staff time for research, coordination of research and clinical activities, and educating providers/staff about research studies could improve cancer-related research activities in persistent poverty areas.

## Introduction

Despite advances in outcomes across the cancer control continuum, residents of persistent poverty areas, which are overwhelmingly rural (85%) [1] continue to face significant cancer health disparities [2–5]. Persistent poverty areas are defined as places where 20% or more of residents have lived in poverty for the past 30 years [3, 4]. Based on 1990–2020 data sources, there are 354 persistent poverty counties in the United States (U.S.), representing 11.3% of counties nationwide [6]. Persistent poverty areas have high concentrations of racial minorities and are primarily concentrated (nearly 80%) in Southern states [3, 4]. Persistent poverty is a product of intersecting structural factors, including economic disinvestment, structural racism, and residential segregation [2, 4, 7, 8]. Residents of these areas face high unemployment, low educational attainment, lack of adequate housing, and high rates of food insecurity and stress [1, 3, 9]. They have also endured decades of disinvestment in medical infrastructure, resulting in pronounced barriers to healthcare access [1, 3].

Cancer risk is significantly elevated, and cancer outcomes are significantly poorer, among people living in persistent poverty areas [3, 4]. On average, populations living in persistent poverty areas have higher rates of known risk factors for cancer, including obesity, tobacco use, alcohol consumption, sun exposure, and human papillomavirus infection [3, 10]. In addition, chronic exposure to social and economic disadvantage may lead to accelerated epigenetic aging and increased susceptibility to cancer [11, 12]. Persistent poverty areas have among the highest cancer mortality rates in the country [3]. Overall cancer mortality in persistent poverty counties is 12% higher than all other U.S. counties and 7% higher than other counties experiencing current (but not persistent) poverty [3]. Notably, the highest cancer mortality rates were found among Black residents of rural, persistent poverty counties, overall and for multiple specific cancer sites [4].

**Fig. 1:**
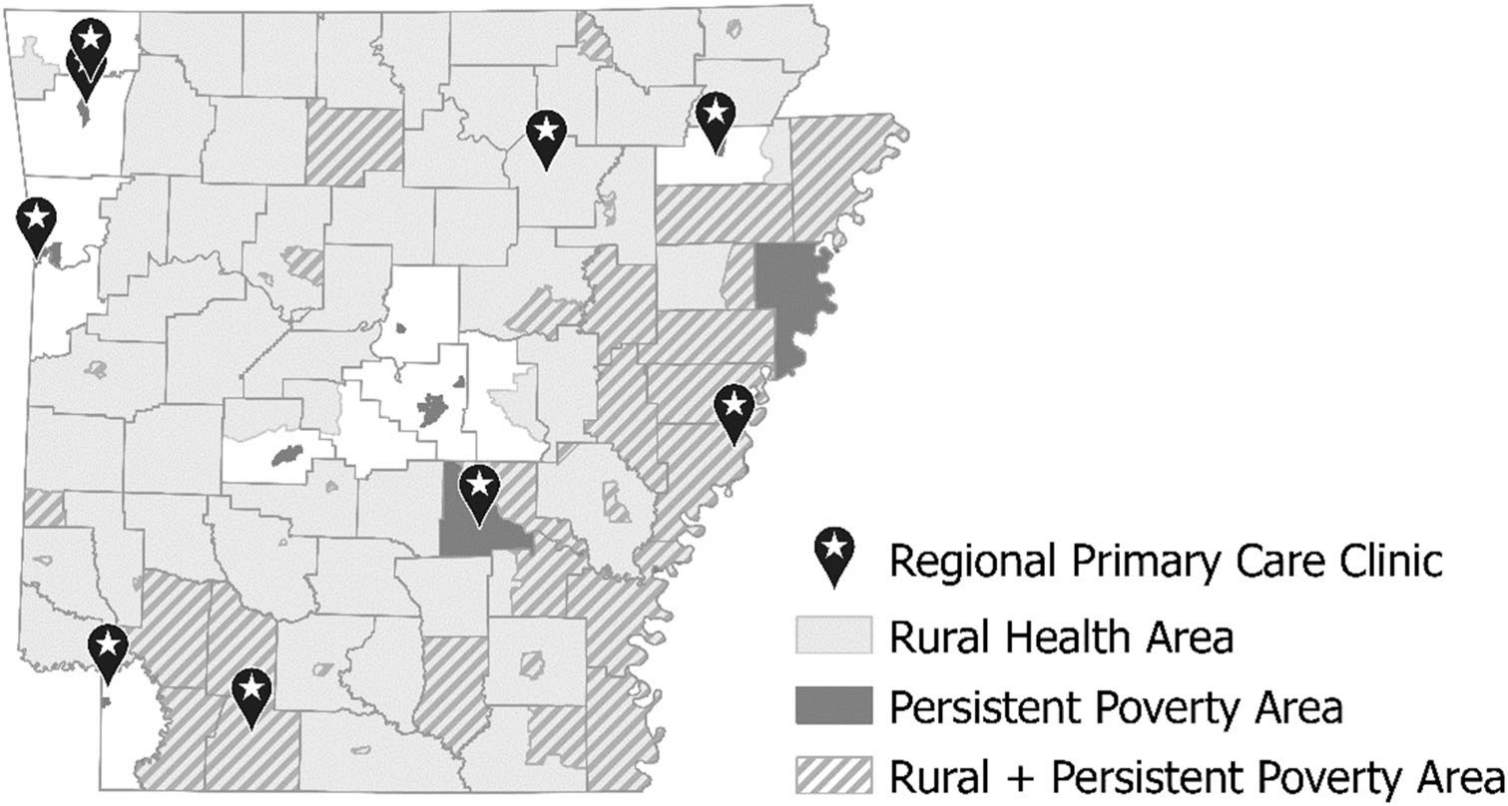
Persistent poverty areas and rural health areas, combined county and census-tract levels, and primary care clinic locations. *Data Source* Economic Research Service, USDA (2022) [16]; HRSA Rural Health Areas (2021) [21]

Arkansas is a disproportionately rural and impoverished state. Over 41% of the population lives in rural areas compared to 14% in the United States (U.S.) overall [13, 14], and 16.3% live in poverty compared to 11.6% in the U.S. overall [15]. In Arkansas, there are 17 persistent poverty counties, plus 62 census tracts in 26 additional counties designated as persistent poverty areas [16]. These areas are located throughout the state but are most concentrated in the southern and eastern regions of Arkansas along the Mississippi River Delta (see Fig. 1).

The National Cancer Institute (NCI) has called for increased attention and research to improve cancer health equity in persistent poverty areas [1, 2]. The Centers for Disease Control and Prevention and the National Academies of Sciences, Engineering, and Medicine have similarly called attention to the stark health disparities affecting rural and persistent poverty communities; they have called for action to improve health outcomes among these communities [17–20]. Increased uptake of evidence-based cancer prevention and control strategies could mitigate cancer health disparities in persistent poverty areas [2]. However, more research is needed on the contextual determinants that may influence the strategic implementation of such strategies. To identify these contextual determinants, it is imperative to understand the barriers, facilitators, and priority needs related to cancer prevention, control, and research in persistent poverty areas. In this study, we conducted focus groups with providers and staff at eight primary care clinics serving persistent poverty areas across Arkansas. Our goal was to gain on-the-ground knowledge and insight from healthcare providers and clinic staff members who work daily with populations living in persistent poverty areas. Our results may help inform opportunities to adapt and implement evidence-based interventions to improve cancer prevention, control, and research in persistent poverty areas.

## Methods

### Recruitment, inclusion criteria, and remuneration

The study team created a flyer that was advertised on university system Listservs and sent directly to the administrative personnel at eight primary care clinics serving persistent poverty areas throughout the state. Participants were eligible if they were current health care providers or staff members at one of the eight primary care clinics. All individuals who replied to express interest in the study and who were eligible to participate were included in the study. Each participant was offered a $40 gift card as renumeration for their participation; two participants declined the remuneration.

### Data collection

Three separate focus groups were conducted in September 2022. Each focus group was held virtually to easily convene conversations among providers and staff located at different clinics across the state. Each focus group was facilitated by one of two researchers with expertise in qualitative methodology and community-based participatory research. Two research coordinators were also present at each focus group to coordinate logistics, keep track of attendance, and take notes but did not participate in the discussions. The number of participants ranged from three to eight in each group. The duration of the focus group discussions ranged from 35 to 60 min.

### Study instruments

The study team used a qualitative interview guide that was created in collaboration with a 19-member Community Advisory Board (CAB) composed of community leaders representing rural and persistent poverty areas across the state. The interview guide was developed and refined through an iterative review process between the CAB and study team. Participants also completed a brief questionnaire that included participant demographic information, clinic location, and their role at their clinic.

### Data analysis

Each focus group was audio-recorded and transcribed by a third-party service. The first and second authors compared the audio recordings of the focus groups to the transcripts multiple times to ensure accuracy and make corrections as needed. Two researchers with expertise in qualitative analysis (EH and KHKY) separately coded each transcript. The two researchers met three times to compare initial codes, develop a codebook with definitions and example codes, and refine themes and codes until they reached inter-coder agreement. The codebook with themes and sub-themes was also reviewed and confirmed by the two qualitative researchers who facilitated the focus group discussions (RSP and PAM). In addition, an administrative director of research (BJW) who is based at a primary care clinic in a rural, persistent poverty county reviewed the themes and sub-themes and provided constructive feedback. This feedback was incorporated into the final organization and presentation of the results. The first author kept detailed meeting notes to keep track of analytic decisions during data analysis.

To present the results clearly and succinctly, Tables 1 and 2 describe the themes and sub-themes with representative quotes. Each quotation has a descriptor that includes the participant’s focus group number (e.g., FG1 = focus group 1) and their role at their primary care clinic.

**Table 1:** Barriers, facilitators, and priority needs for cancer control and prevention services at primary care clinics serving rural persistent poverty areas

Theme	Theme description	Exemplary quote(s)
*Barriers*		
Transportation	Patient transportation services are limited and often unreliable (e.g., public, paratransit, private). Some patients are unable to drive themselves or find a ride, which makes transportation a significant barrier.	“The problem in Jonesboro […] is a lot of people have to depend on [public transit], [or] on Medicaid vans […], which aren’t always on time. […] It is a real struggle for those that don’t have their own transportation or someone that can take them to these appointments, [which is] a large number of our population.” [FG3, Administrator] “In our area, especially for those who have [Medicare] Advantage Plan, a lot of them are switching to using Uber. A [patient] had an appointment, but 15 min before [their] appointment, the Uber called and cancelled…We have to have someone who is committed and dedicated to taking our patients to the doctor.” [FG3, Navigator]
Medical costs	Cost is a major barrier for many low-income patients, who are reluctant to engage in positive health behaviors if there is a cost.	“When I first went [for a colon cancer screening], I had to pay $1,500 up front. I didn’t have it so I didn’t get the screening done. A lot of my patients, if it costs just a little bit, they’re not going to do it. […] The financial part was a big issue for me and it’s a big issue for a lot of my patients.” [FG2, Nurse]
Limited providers and service availability	Limited provider time to discuss prevention with patients, lack of providers in rural areas, and limited service days at rural hospitals and primary care clinics are barriers to cancer control and prevention among patients in persistent poverty areas.	“I feel like we're depending heavily on the providers to offer the opportunity [for cancer screening]. But they're only given a short amount of time to see patients. By the time they get through addressing the acute issue, the patient is out the door. They didn't really get a chance to address care gaps.” [FG2, Quality Improvement Coordinator]
Patient fear or discomfort with cancer-related topics and knowledge	Patients’ fear and/or discomfort with cancer-related topics, or even with knowledge about their own cancer diagnosis, are barriers to providing both cancer prevention and treatment.	“Some people don't even want to know the outcomes of their screening because they're dealing with so much trauma and stress already in their daily life that they just can't even think of adding another thing that's as big as cancer.” [FG1, Administrator] “As far as colon cancer is concerned, our patients are not comfortable with even handling their own [stool] to even do that test. So, if we could get more outreach, maybe that would help normalize that that's not something that is too far out of reach for them. […] When it comes to colonoscopy, we have local providers here, but they're always so booked up it takes a long time to get the appointment. So, by the time [of their appointment], they kind of talk themselves out of doing it [and] don't keep their appointment. Or, they go to the initial appointment for the consultation, but don’t keep the colonoscopy appointment. [The problem is] just getting them to buy into it and proceed with [screenings].” [FG2, Quality Improvement Coordinator]
*Facilitators*		
Cancer navigators	Cancer navigators are key facilitators in connecting patients with cancer prevention services, care, and research opportunities and could make a significant difference in care access for patients. Cancer navigators have the skills and rapport with patients required to close care gaps. Navigation services could be improved if cancer navigators had more support and resources.	“Because cancer is a really scary diagnosis, and you need someone [help someone navigate diagnosis and treatment], [and] if they can't be that advocate for themselves, they need someone that can be that advocate for them.” [FG3, Administrator] “We sent out a lot of automated messages and reminders that are ignored by our patients and having that personal touch with patient navigators is just essential. I'm seeing a lot of benefit with that.” [FG3, Data Quality Team]
Community health events and services	Community health events and services are catalysts that can nudge a person toward a positive health behavior (e.g., a screening test) *in-the-moment.* Offering in-the-moment opportunities through community health services was stated as an important way of providing health care access to low-income patients unable to schedule cancer prevention screenings.	“I did feel that the health fairs that we were having in our community were somewhat helpful, especially those that were offering free screenings. I did have men that would ask me, ‘At this fair, are they going to offer the free prostate type screening or exams?’” [FG2, Quality Improvement Coordinator] “If you have a health fair, or something like the blood mobile… a lot of times somebody will think about that in the moment and go sign up to give blood. What if that free [mobile mammography unit] was out there, and you decide, my doctor's been after me, I might want to get that done.” [FG2, Administrator]
*Priority needs*		
Patient education	Providing patient education about the importance of cancer screenings would facilitate greater patient engagement with cancer prevention and control services.	“Patients don't seem to understand the true need for [mammograms], and how that can actually save their lives.” [FG3, Administrator] “[I think that nurses and other health care workers should spend] more time talking to the patient about how important [screening] is. Not just going in, [getting their] vitals, leave. But like, you really need to talk about this, even if they don't have cancer.” [FG1, Medical Lab Scientist] “[We need to] get our patients better educated. Our initial contact with the patient is calling, introducing myself, and saying, ‘Hey, I see you haven’t got your mammogram in the past 4 or 5 years. Have you had it? But just [not] with us?’ I had one lady say, ‘Oh, I’m not going to get that! That’s hurts.’ Then that opens the door for me to educate her on that.” [FG3, Navigator]
Comprehensive workflows	A comprehensive work flow that all providers, staff, and clinics are aware of and work together to implement and follow is needed to ensure patients do not fall through the cracks and are successfully navigated across the cancer continuum.	“[We need] some type of [screening] algorithm, so that no matter what [a patient’s] preference is, or no matter what barriers we meet, there’s another plan to try to help her. [For example,] if the navigator calls and tries to schedule, ‘I realize that you haven't had your colonoscopy. Can I send you to the front desk to get your colonoscopy scheduled?’” [FG1, Navigator]
Improved communication	Improved communication between primary care clinics and the Cancer Institute and better systems for sharing medical records would facilitate cancer prevention and control services.	“One practical thing [to improve cancer control and prevention programs in our clinics] is to have a communication line between our clinics and [the Cancer Institute]. We need a point of contact [with them]. We need to all collaborate to make sure everybody's on the same page and trying to reach the same goal.” [FG3, Navigator] “[During a call with a patient] I said, ‘it looks like it’s been past ten years [since your last colorectal cancer screen].’ He said ‘No, I had it three years ago.’ […] We don't have a good way to get all of our records in house, because a lot of the other facilities don't share.” [FG1, Navigator]
Integrate navigators into healthcare team	Better integration of navigators into the healthcare team (i.e., improved understanding of their role among clinicians, increased referrals, increased access to medical records) to maximize their potential to close care gaps and improve patient care would better facilitate cancer prevention and control services.	“I think our cancer navigators are a great resource, and [I’d like] for us to intentionally integrate them into the care team there in the clinic.” [FG1, Data Quality Team] “We need the buy-in of the providers. Because I feel that if the provider has a patient that has cancer, and when they get that diagnosis at first, if they could just refer them to us (navigators), then we can reach out and touch them in the beginning. Help them maneuver through the whole process. Give us the opportunity to talk with the patient and let them know what we can do.” [FG3, Navigator] “What we're doing now is waiting ‘til we get access to EPIC (medical records database). We are putting notes in there, but we need access to EPIC, so a provider or someone will know, a navigator went in and talked to this person.” [FG3, Navigator]

**Table 2:** Barriers and facilitators related to cancer-related research at primary care clinics serving rural persistent poverty areas

Theme	Theme description	Exemplary quote(s)
*Barriers*		
Lack of time	Primary care clinic providers and staff are open to assisting with cancer-related research but lack the time to support research efforts.	“I think we would be open to help with any kind of research that's cancer-related. The problem is that being in a residency program, the time constraints of everybody involved is probably the biggest barrier. It would have to be something we could get dedicated time to help with because our faculty are stretch pretty thin already.” [FG3, Administrator]
Community uncertainty, skepticism	Patients sometimes have uncertainty or skepticism toward cancer-related research, because they often do not understand the purpose of the research or what participation entails and are not aware of the outcomes or benefits of the research for their community.	“A lot of communities are just uncertain, nervous, or scared. They don't quite understand, so that may be preventing them from joining the study.” [FG1, Research Coordinator] “[Researchers need] a very serious interest about making a change in the community. Because we have people that come into the community. They want to do the surveys, but when it comes down to it, what are the outcomes? [The community doesn’t] know what the outcomes are. They're not seeing that difference in their community that they need to see.” [FG1, Director]
Health literacy	Health literacy is a barrier to cancer research in persistent poverty areas. The language of study information and documents (e.g., consent document) needs to be plain so the patient can fully understand and feel confident taking part in a research study.	“[One contributor] to some of the various [problems] in our rural community is […] literacy. […] I know that oftentimes we’ve found ourselves having to explain in layman's terms, or below layman's terms, if that's a word. For them to truly understand what they're agreeing to, or consenting to.” [FG1, Director]
Provider skepticism, patient burden concerns	Concerns about study design and implementation may be a barrier to increased cancer research in these areas. Projects need to be collaboratively planned to reduce patient and clinical burdens or disruptions.	“I think that there will be some skepticism with working with research because of a process that went wrong a while ago. […] Some diabetes researchers [came into] the family medicine clinic [and collected] a blood stick for hemoglobin. [However,] that that information was stored with research and was not cross-compatible with family medicine. Those patients subsequently refused finger sticks once they got into their actual appointment because we were not able to use [the first finger stick for] both. […] that was obviously a deterrent for getting cooperation from patients.” [FG3, Data Quality Team]
*Facilitators*		
Inform clinic providers/staff about research studies	Educating primary care clinic providers and staff about current studies their patients may be eligible for and providing information to promote research opportunities could facilitate more patient participation in cancer research.	“[I would recommend] a presentation of some sort to the clinic staff as a whole. So that they're aware [of the study]. That way they can get on board and feel involved. Then, when they encounter a patient that may be eligible to participate, they can do that referral, and feel good about that.” [FG 1, Research Coordinator]
Navigators as a bridge between the clinic and patients	Cancer navigators could serve as a bridge to help clinic staff connect patients with cancer-related research opportunities.	“One of the [important] things is introducing the clinic personnel to the navigators. Connecting that partnership, so that when these types of services are needed, they know that they have a navigator at that clinic, and what they are able to assist with in support of these research studies.” [FG1, Director]

## Results

### Sample description

A total of 17 providers and staff, representing all eight primary care clinics across the state, participated in three focus groups. Participants had a diverse range of roles at their clinics, including administrators, nurses, navigators, research coordinators, data quality team members, and others. Nine participants identified as white, four identified as Black or African American, one preferred not to answer, and three participants had missing racial/ethnic group data. Fourteen participants identified as female, and three identified as male.

### An underlying theme: poverty and high health-related social needs

An underlying theme throughout all three focus groups was how decades of poverty and high levels of health-related social needs facing patients in persistent poverty areas shape healthcare providers’ work at primary care clinics across the state. As one participant summarized, “Generational poverty and all the social drivers of health that come along with that, that we've got in our state, kind of make it so you've got clusters of problems. It’s not like people just need food or just need transportation. You have people who need everything.” [FG1, Data Quality Team] A healthcare provider explained, “A lot of my patients, if it costs just a little bit, they’re not going to do it. […] The financial part [is] a big issue for a lot of my patients.” [FG2, Nurse] Another participant noted, “Our population here, we have a lot of Medicaid patients, so one of the biggest struggles for them is transportation.” [FG3, Administrator] Some participants emphasized that these issues were not new but were long-standing barriers that affect access to healthcare and the health of their patient populations generally. As one administrator noted, “You know, it's the same [barriers] we've been seeing for many, many years. I mean, when you look at your colonoscopies, your mammograms, a lot of the barriers are the same.” [FG1, Administrator] While daunting, leveraging clinic providers’ and staff’s deep understanding of the long-standing and interrelated health-related social needs their patients face can be instructive for identifying solutions and interventions that can be broadly applied among patients in persistent poverty areas, e.g., strategies need to comprehensively address poverty and social determinants of health to make a meaningful impact in persistent poverty areas. Interventions aimed at more equitable health care access must address the clusters of problems instead of just one barrier at a time (e.g., transportation).

Below, we discuss two interrelated sets of findings based on discussions with providers and staff at primary care clinics. First, we discuss the barriers, facilitators, and priority needs related to cancer prevention and control described by providers and staff at primary care clinics. Second, we discuss the barriers and facilitators to conducting cancer-related research at primary care clinics described by clinic providers and staff.

### Barriers, facilitators, and priority needs related to cancer prevention and control

In Table 1, we provide descriptions and exemplary quotes of the barriers, facilitators, and priority needs related to cancer prevention and control that providers and staff encounter at primary care clinics serving the state’s persistent poverty areas. We identified multiple *barriers* to cancer prevention and control programs at their clinics, including transportation, medical costs, limited providers and service availability, and patient fear or discomfort with cancer-related topics and knowledge. Participants described two key *facilitators*: cancer navigators and community health events and services. While participants discussed fewer facilitators, they described both as having great potential to positively impact the health of their patient populations. *Priority needs* were a significant topic of interest in all focus groups. Providers and staff identified the following as priority needs to improve cancer prevention and control programs: patient education, comprehensive workflows, improved communication, and integration of cancer navigators into healthcare teams.

### Barriers and facilitators of cancer-related research

In Table 2, we provide descriptions and exemplary quotes of the facilitators and barriers to cancer-related research identified by clinic providers and staff. *Barriers* to cancer-related research at clinics included lack of time, community uncertainty/skepticism of research’s benefits, health literacy, and provider skepticism of research/patient burden concerns. *Facilitators* included informing clinic providers/staff about research studies and leveraging cancer navigators as a bridge between clinics, patients, and researchers. These were discussed as *potential* facilitators that, if implemented, could enable increased cancer-related research efforts at the clinics.

While focus group discussions focused primarily on barriers to cancer-related research, with less attention to facilitators, clinic partners were clear that they were not opposed to integrating and increasing research activities at their clinics. Rather, they explained it was a matter of creating the right conditions and providing resources to support clinic-based research. For example, one community partner noted lack of time as a barrier but suggested providing the primary care clinics with dedicated staff time to help support research activities.

## Discussion

The findings of this study can help inform the adaptation and implementation of evidence-based strategies to improve cancer prevention, control, and research in persistent poverty areas. We identified multiple *barriers, facilitators, and priority needs* relevant to work addressing cancer inequities in these geographical areas. The barriers that clinic providers and staff described align with previous work on barriers to cancer prevention and control in rural and impoverished communities [5, 22–26]. Barriers including lack of transportation, medical costs, and limited availability of cancer-related health services have been documented in past research on rural cancer disparities [5, 22–25]. Patient fear of cancer screening tests, test results, and cancer itself have been documented in multiple studies on barriers to cancer screening uptake among rural, poor/low-income, and other underserved communities [26–30].

Participants identified two key facilitators to improve cancer health outcomes. Cancer navigators were identified as facilitators of cancer prevention and control activities, reflecting the increasing recognition of patient navigation as a key element in high-quality, patient-centered cancer care. This is consistent with prior literature which documents the value of cancer navigators in cancer prevention and survivorship care, as well as in rural settings, communities living in poverty, and other underserved communities [31–36]. Clinic providers and staff also identified community health events and services as a facilitator, which is consistent with prior literature demonstrating the efficacy of a range of community-based cancer outreach and screening interventions [37–41].

Critical priorities for cancer prevention and control in persistent poverty areas included patient education, which is consistent with prior research highlighting the beneficial effects of tailored and/or personalized education in facilitating patient engagement, especially for uptake of screenings [42–45]. Comprehensive workflows and improved communication were also identified as priority needs and are consistent with research demonstrating the need for improved cancer care coordination to improve outcomes across the cancer control continuum [46–48]. The identified need to fully integrate cancer navigators into cancer care teams aligns with work that has recommended including navigators as members of the clinical team to optimize patient outcomes [49].

We also identified *barriers and facilitators* to cancer-related research at primary care clinics serving persistent poverty areas. The barriers, including lack of time, community uncertainty/skepticism, health literacy, and provider skepticism/patient burden concerns, align with prior work about research participation barriers in underserved communities [50, 51]. Clinic-level facilitators to research were also consistent with previous research [52] that included informing clinic providers/staff about research studies and leveraging cancer navigators to conduct research.

Based on the study’s findings, we have developed several recommendations to improve cancer outcomes in persistent poverty communities. These recommendations will be used by the clinics and authors to improve cancer outcomes in persistent poverty areas of Arkansas, and they have broader implications for providers and healthcare systems serving persistent poverty areas throughout the U.S. The recommendations to improve cancer prevention and control in persistent poverty areas are: (1) develop locally-informed resources to reduce patient transportation and cost barriers; (2) increase patient education efforts; (3) standardize patient navigation workflows; (4) better integrate cancer navigators into healthcare teams; and (5) use community health events as key healthcare access points. Recommendations to improve cancer-related research in persistent poverty areas include: (1) provide dedicated staff time for research activities at clinics; (2) coordinate research and clinical activities to avoid patient burden and clinic disruptions; and (3) educate providers and staff about research studies so they can knowledgably inform patients about studies for which they may be eligible. Notably, regional clinics’ and our healthcare system’s ability to act on these recommendations will require significant investment and resources.

The recommendations proposed above will require multilevel interventions on the individual level (e.g., improve patient education/knowledge), systems level (e.g., better integrate cancer navigators into healthcare teams), and structural level (e.g., improve local transportation infrastructure). Others have made similar calls for multipronged interventions at the individual, systems, and structural levels to reduce cancer health inequities experienced by communities living in poverty [53], including persistent poverty communities [2].

Overall, it is critical that efforts to address cancer health disparities in persistent poverty areas leverage the knowledge and resources in those communities. While many of the findings are consistent with prior literature, this is one of the first qualitative studies to document these barriers, facilitators, and priority needs as described by providers and clinic staff in persistent poverty communities. Gaining this information specific to persistent poverty communities is critical for engaging stakeholders in developing community-informed, locally relevant interventions. Efforts to address these disparities in persistent poverty communities are constrained because many places with high concentrations of persistent poverty areas, including Arkansas, do not have an NCI-designated and funded cancer center [54]. This lack of investment results in a systematic widening of disparities.

### Strengths and limitations

Study strengths include sampling providers and staff from primary care clinics serving persistent poverty areas throughout Arkansas to ensure the representation of state-wide perspectives. This study also identified facilitators and barriers at the healthcare system level to cancer prevention and control specifically within persistent poverty communities, who bear a disproportionate cancer burden. Though this qualitative study included a relatively small sample, examination of the data indicated that data saturation had been reached. While this study was conducted in Arkansas, the findings may inform future efforts to ascertain how to best address cancer inequities in persistent poverty areas across the country. Future research in this area should engage patients and their caregivers directly to understand the barriers, facilitators, and priority needs to improve cancer outcomes from their perspectives. Further, the findings from this study should be leveraged in future work to adapt and implement evidence-based cancer prevention and control strategies in persistent poverty areas.

## Conclusion

Persistent poverty has long been a multifaceted and intractable social ill. Effectively addressing and reducing the cancer health inequities suffered by residents of persistent poverty areas requires substantial financial investment and healthcare systems-level and policy-level efforts. The NCI’s call for increased attention and research on areas experiencing persistent poverty has provided an opportunity to illuminate the complex determinants of persistent poverty, its resulting cancer health inequities, and how to develop strategic, community-informed interventions to address them. We have provided practical recommendations based on the results of this study that healthcare systems can act on to improve cancer prevention, control, and research in persistent poverty areas.

## Data Availability

Not applicable.

## References

[1] National Cancer Institute (2022) Persistent poverty and cancer: increasing health equity across the cancer continuum. https://www.cancer.gov/research/annual-plan/scientific-topics/increasing-health-equity#:~:text=Persistent%20poverty%20is%20defined%20as,multifaceted%20nature%20of%20this%20problem. Accessed 15 Sept 2022

[2] Gomez SL, Shariff-Marco S, Cheng I (2022). The unrelenting impact of poverty on cancer: structural inequities call for research and solutions on structural determinants. J Natl Cancer Inst.

[3] Moss JL (2020). Persistent poverty and cancer mortality rates: an analysis of county-level poverty designations. Cancer Epidemiol Prevent Biomark.

[4] Moss JL (2022). Enduring cancer disparities by persistent poverty, rurality, and race: 1990–1992 to 2014–2018. J Natl Cancer Inst.

[5] Yabroff KR (2020). Rural cancer disparities in the United States: a multilevel framework to improve access to care and patient outcomes. JCO Oncol Pract.

[6] Dalaker J (2022) The 10–20–30 provision: defining persistent poverty counties. Congressional Research Service

[7] Miller CE, Vasan RS (2021). The southern rural health and mortality penalty: a review of regional health inequities in the United States. Soc Sci Med.

[8] Harris RP, Worthen D (2003) African Americans in rural America. Challenges for rural America in the twenty-first century. Penn State University Press, Pennsylvania, pp 32–42

[9] Champagne CM (2007). Poverty and food intake in rural America: diet quality is lower in food insecure adults in the Mississippi Delta. J Am Diet Assoc.

[10] Crosby R (2012). Rural populations and health: determinants, disparities, and solutions.

[11] Warnecke RB (2008). Approaching health disparities from a population perspective: the National Institutes of Health Centers for Population Health and Health Disparities. Am J Public Health.

[12] Alcaraz KI (2020). Understanding and addressing social determinants to advance cancer health equity in the United States: a blueprint for practice, research, and policy. Cancer J Clin.

[13] Dobis E et al (2021) Rural America at a glance: 2021. Economic Research Service, USDA

[14] Miller W, Wheeler E (2021) Rural profile of Arkansas: social & economic trends affecting rural Arkansas. University of Arkansas, Division of Agriculture Research and Extension, Fayetteville. p 7

[15] United States Census Bureau (2022) QuickFacts Arkansas. https://www.census.gov/quickfacts/AR. Accessed 20 Feb 2023.

[16] Economic Research Service (2022) Data from: poverty area measures. http://ers.usda.gov/data-products/poverty-area-measures. Accessed 24 Mar 2023.

[17] National Academies of Sciences, E. and Medicine (2021) Population Health in rural America in 2020: proceedings of a workshop. The National Academies Press, Washington, p 154. 34748300

[18] Garcia MC (2020). Bridging the gap in potentially excess deaths between rural and Urban Counties in the United States. Public Health Rep.

[19] O’Neil ME (2019). Lung cancer incidence in nonmetropolitan and metropolitan counties—United States, 2007–2016. Morb Mortal Wkly Rep.

[20] Henley SJ, Jemal A (2018). Rural cancer control: bridging the chasm in geographic health inequity. Cancer Epidemiol Biomark Prev.

[21] Federal Office of Rural Health Policy (2021) Data from: list of rural counties and designated eligible census tracts in Metropolitan Counties. http://data.hrsa.gov/tools/rural-health. Accessed 24 Mar 2023

[22] Ratnapradipa KL (2022). Qualitative analysis of cancer care experiences among rural cancer survivors and caregivers. J Rural Health.

[23] Charlton M (2015). Challenges of rural cancer care in the United States. Oncology (Williston Park).

[24] Wercholuk AN, Parikh AA, Snyder RA (2022). The road less traveled: transportation barriers to cancer care delivery in the rural patient population. JCO Oncol Pract.

[25] Lee KMN (2023). Distance and transportation barriers to colorectal cancer screening in a rural community. J Prim Care Community Health.

[26] Jones RM (2010). Patient-reported barriers to colorectal cancer screening: a mixed-methods analysis. Am J Prev Med.

[27] Garbers S (2003). Barriers to breast cancer screening for low-income Mexican and Dominican women in New York City. J Urban Health.

[28] Gesink D (2016). Cancer screening barriers and facilitators for under and never screened populations: a mixed methods study. Cancer Epidemiol.

[29] Tessaro I (2006). Knowledge, barriers, and predictors of colorectal cancer screening in an Appalachian church population. Prev Chronic Dis.

[30] Vapiwala N (2021). Stigma, beliefs and perceptions regarding prostate cancer among Black and Latino men and women. BMC Public Health.

[31] Dohan D, Schrag D (2005). Using navigators to improve care of underserved patients. Cancer.

[32] Kline RM (2019). Patient navigation in cancer: the business case to support clinical needs. J Oncol Pract.

[33] Bernardo BM (2019). The efficacy and cost-effectiveness of patient navigation programs across the cancer continuum: a systematic review. Cancer.

[34] Robinson-White S (2010). Patient navigation in breast cancer: a systematic review. Cancer Nurs.

[35] Guide to Community Preventive Services (2023) CPSTF recommends patient navigation services to increase cancer screening and advance health equity. https://www.thecommunityguide.org/news/cpstf-recommends-patient-navigation-services-increase-cancer-screening-advance-health-equity.html. Accessed 24 Jan 2023

[36] Rohan E (2022). Pairing Project ECHO and patient navigation as an innovative approach to improving the health and wellness of cancer survivors in rural settings. J Rural Health.

[37] Greenwald ZR (2017). Mobile screening units for the early detection of cancer: a systematic review. Cancer Epidemiol Biomark Prev.

[38] Mason TA (2013). Evaluation of the Avon foundation community education and outreach initiative community patient navigation program. Health Promot Pract.

[39] Murray K (2014). The reach and rationale for community health fairs. J Cancer Educ.

[40] Pasick RJ, Hiatt RA, Paskett ED (2004). Lessons learned from community-based cancer screening intervention research. Cancer.

[41] Schoenberg NE, Howell BM, Fields N (2012). Community strategies to address cancer disparities in Appalachian Kentucky. Fam Community Health.

[42] Chelf JH (2001). Cancer-related patient education: an overview of the last decade of evaluation and research. Oncol Nurs Forum.

[43] Conley CC, Ryba MM, Andersen BL, Duckworth MP, O'Donohue WT (2018). Behavioral health and cancer. Behavioral medicine and integrated care.

[44] Robb KA (2021). The integrated screening action model (I-SAM): a theory-based approach to inform intervention development. Prevent Med Rep.

[45] Tolotti A (2022). Patient engagement in oncology practice: a qualitative study on patients’ and nurses’ perspectives. Int J Environ Res Public Health.

[46] Paladino J (2019). Evaluating an intervention to improve communication between oncology clinicians and patients with life-limiting cancer: a cluster randomized clinical trial of the serious illness care program. JAMA Oncol.

[47] Penedo FJ (2020). The increasing value of eHealth in the delivery of patient-centred cancer care. Lancet Oncol.

[48] Gorin SS (2017). Cancer care coordination: a systematic review and meta-analysis of over 30 years of empirical studies. Ann Behav Med.

[49] Blackley K (2021). Cancer patient navigation. BMJ Support Palliat Care.

[50] Salman A (2016). A review of barriers to minorities' participation in cancer clinical trials: implications for future cancer research. J Immigr Minor Health.

[51] Schmotzer GL (2012). Barriers and facilitators to participation of minorities in clinical trials. Ethn Dis.

[52] Nipp RD, Hong K, Paskett ED (2019). Overcoming barriers to clinical trial enrollment. Am Soc Clin Oncol Educ Book.

[53] Sayani A (2021). Advancing health equity in cancer care: the lived experiences of poverty and access to lung cancer screening. PLoS ONE.

[54] National Cancer Institute (2019) NCI-designated cancer centers. Cancer research infrastructure. https://www.cancer.gov/research/infrastructure/cancer-centers. Accessed 1 Mar 2023

